# Experiences and perspectives of women who have committed neonaticide, infanticide and filicide: A systematic review and qualitative evidence synthesis

**DOI:** 10.1111/jpm.12828

**Published:** 2022-03-24

**Authors:** Giulia Milia, Maria Noonan

**Affiliations:** ^1^ Faculty of Education and Health Science University of Limerick Limerick Ireland

**Keywords:** child killing, filicide, infanticide, maternal mental health, neonaticide, qualitative systematic review, women's experiences

## Abstract

**What is known on the subject?:**

- The phenomenon of child killing (neonaticide, infanticide or filicide) is a rare event that cannot be fully explained by a single construct as each case involves the unique life circumstances of each woman who committed the act(s).

**What the paper adds to existing knowledge?:**

- The majority of women who committed neonaticide, infanticide or filicide regretted the act and regretted not seeking help from family and healthcare professionals.
- Women who committed neonaticide, infanticide or filicide in the main had complex circumstances characterised by poverty, abusive relationships, poor family and social support or over reliance on family supports and mental health issues.

**What are the implications for practice?:**

- Women require a clear plan of what to do if they feel overwhelmed with caring for a baby or child.
- Healthcare professionals involved with women in the perinatal period need to explore further women's expressions of “not being ready to be a mother” which for some women may be pathological and require further assessment.
- Women need to be made aware of the support service pathways available to them during the perinatal period and beyond.
- Further research is needed to explore and learn from women's experiences to reduce child homicide mortality and support women and their families.

**Abstract:**

**Introduction:**

Meaning and personal experiences of the acts of neonaticide, infanticide and filicide have rarely been investigated from the perspectives of the women who committed those acts.

**Aims:**

To identify and synthesise evidence on the perspectives of women directly involved in the complex phenomena of neonaticide, infanticide or filicide from the evidence available on their unique point of view and how these experiences have affected women's lives. To understand how the experiences and perceptions of women who engaged in child killing present similarities or differences according to the child's age at time of death.

**Methods:**

Qualitative primary studies published in English were included if they explored the experiences of women who engaged in neonaticide, infanticide or filicide. Methodological quality was assessed using the qualitative Critical Appraisal Skills Programme (CASP) checklist. A thematic analysis framework guided the synthesis.

**Results:**

Seven papers reporting on five studies met the inclusion criteria for the review. Three analytical themes were identified: Not ready to be a mother; Intentionality and premeditation in the context of trauma and mental health issues; Sorrow of regret.

**Discussion:**

The majority of women who committed neonaticide, infanticide or filicide had complex psychological, social and personal circumstances and in the main regretted the act and regretted not seeking help from family and healthcare professionals. Healthcare professionals in contact with women during the perinatal period and beyond need to be aware of the profiles of vulnerable women and undertake holistic integrated assessments to identify the woman's personal context, changes in interpersonal relationships, social isolation or over reliance on family supports and changes in mental health status or new onset of mental health conditions.

**Implications for Practice:**

Women require a clear plan of what to do if they feel overwhelmed with caring for a baby or child. Healthcare professionals involved with women in the perinatal period need to explore further women's expressions of “not being ready to be a mother” which for some women might be pathological and require further assessment. Women need to be made aware of the support services pathways available to them during the perinatal period and beyond. Further research is needed to explore and learn from women's experiences of each of the phenomena separately to reduce child homicide mortality and support women and their families.

## 1 INTRODUCTION

A sense of social taboo around the rare phenomenon of child killing is evident in every society. A probable explanation for this could be the common and shared idea across continents that parents, mothers in particular, are by nature programmed to be caring and protective towards their children. This means that when they engage in acts of neonaticide, infanticide or filicide, they confound the socially accepted norms and their actions become intolerable and inexplicable to others (Klier et al., 2019).

The worldwide incidence of neonaticide, infanticide or filicide varies across countries. Findings from the global United Nations on Drugs and Crime (UNODC, 2019) where child homicide data from 41 countries are included found that in 2016 the homicide rate of those aged 0–19 years was 2.6 per 100,000 population, with US figures consistently higher (5.3 per 100,000 population) in comparison with the rest of the world's rates (0.6 per 100,000 population in Europe). Existing global data on child killing were systematically analysed (Stöckl et al., 2017). Figures on filicide of children under 18 were available for 35 countries. Only in 33 countries was a distinction made between maternal and paternal filicide, where mothers were found responsible in 54.7% of filicide cases. Mothers were also found to be involved in infanticide in 71.7% of the cases among 12 countries considered and 100% responsible for neonaticide in 13 countries. Prevalence rates are likely to be underestimated due to: concealment of the crime especially with younger children (Dawson, 2015); difficulties in establishing the real cause of death and discovering the victim's body; scarcity of available data and distinct age breakdowns taken into consideration in different countries when information on children's deaths is collected and reported (UNODC, 2019).

Numerous theories have attempted to rationalise the acts of maternal neonaticide, infanticide or filicide. Women's demographic, historical and clinical traits have been studied along with child victim characteristics to recognise high‐risk women and implement prevention strategies. Although child killing is an ancient practice, the first classification of motives correlated to filicidal or neonaticidal acts was made only in 1969 by Resnick whose model includes five categories: altruistic, acutely psychotic, unwanted child, accidental filicide and spouse revenge (Resnick, 1969). Subsequently, neonaticide was added as a separate category (Resnick, 1970). Although Resnick's model remains influential, it was tailored to a 1960–70s era profoundly different to today's society, especially in terms of stigma, punishment and consequences for extramarital relationships or illegitimacy all of which are now more socially accepted within several cultures (Brown et al., 2019; Meyer et al., 2001; Tanaka et al., 2016). Rarely do those involved in child killing state clearly the motive behind their acts, so classifications based solely on motive might neglect the importance of other variables making a significant contribution to neonaticide, infanticide or filicide (Brown et al., 2019). Nowadays, none of the existing classifications are universally recognised or used to inform prevention due to the high complexity of the acts (Putkonen et al., 2016).

The phenomenon of child killing cannot be fully explained by a single construct as each case involves the unique life circumstances of each woman who committed the act(s); their different contexts and life experiences from childhood until adult life; their personal perceptions of self, the child and others relationships with partner and family (Klier et al., 2019; McKee, 2006). Circumstances and causes relating to neonaticide, infanticide or filicide therefore remain partially unclear and hypothetical, making these acts scarcely predictable (Brown et al., 2019; Friedman et al., 2005).

Though findings can vary significantly according to where the data are drawn from (judicial, coroner, police, prosecution or forensic records), some differences can be noted among traits that characterize the profile of women involved in neonaticide, infanticide or filicide. Women who commit neonaticide are often described as single first‐time young mothers, who have rarely experienced mental health issues, with poor socio‐economic status, living with their parents or other family members, highly prone to conceal or deny their unwanted pregnancy and who avoid both prenatal care and assistance during childbirth (Naviaux et al., 2020).

Similar to those who committed neonaticide, women who have committed infanticide seem to be young, single, with poor education and no access to or scarce attendance at antenatal care (Naviaux et al., 2020). Infanticide is committed in the infant's first year of life—which is described as “the time of peak prevalence for psychiatric illness in women” (Spinelli, 2004). However, the most recent version of the Diagnostic and Statistical Manual of Mental Disorders DSM‐5 (American Psychiatric Association, 2013) does not include postpartum disorders within its formal classification (Spinelli, 2016).

According to the American Psychiatric Association mood disorders “with peripartum onset” involve the “onset of mood symptoms during pregnancy or in the four weeks following delivery with or without psychotic features” and infanticide seems more associated with the latter (American Psychiatric Association, 2013). However, the transition from pregnancy to motherhood can lead women to experience feelings of loneliness, extreme tiredness and isolation, irritability and anxiety due to sudden life changes and increased responsibilities (Razurel et al., 2011) and these feelings can last well beyond the first four weeks after giving birth.

Women within the filicide group seem generally older and more prone to experiencing severe mental health issues which, in different studies (Camperio et al., 2012; Debowska et al., 2015), appear to be a predominant factor relating to maternal filicide. These women often have a history of past or present abuse, addictive disorders and ineffective support systems (Klier et al., 2019; Naviaux et al., 2020).

In the literature, the phenomena of neonaticide, infanticide or filicide are rarely studied solely from a maternal perspective. Other parental figures can be involved, especially in studies where filicide has been included as part of familicide reviews (Karlsson et al., 2019). Familicide cases were defined as the killing of both child and spouse by Aho et al.,(2017) whose systematic review investigated background factors in parents who committed the act of familicide in western families. A different definition of familicide, including filicide of children up to 18 years with or without killing the other spouse or committing suicide, was adopted within Mäkikomsi and Aho's (2018) review, which focuses on understanding the motives for familicide from the perspective of those who committed the acts. The phenomenon of neonaticide has been reviewed from various perspectives with possible predictive factors studied by Friedman and Resnick (2009). Tanaka et al. (2016) documented the worldwide incidence of neonaticide, while Shelton et al. (2011) outlined its characteristics from a law enforcement perspective. Classification of children's fatal maltreatment, risk and protective factors, and strategies of prevention have also been investigated (McCarroll et al., 2017) along with reviews that focused only on cases of filicide towards children with disabilities (Frederick et al., 2019) or congenital abnormalities such as cleft palate (Stewart et al., 2018). The personality and attachment style of filicidal mothers has been explored (Lattanzi et al., 2020) along with possible predictors of maternal filicide such as suicidality and depression or psychosis (Friedman et al., 2005).

## 2 DEFINITIONS OF NEONATICIDE, INFANTICIDE AND FILICIDE

Terms used to define the phenomenon of child killing are neonaticide, infanticide or filicide. Several definitions of neonaticide, infanticide or filicide exist in the literature and are often used interchangeably (Bourget et al., 2007; Debowska et al., 2015) which may lead to a lack of clarity regarding the child's age at time of death or the nature of the relationship between the child and the person who committed the act. The term neonaticide, introduced by Resnick in 1970, is the term most commonly used in the literature and refers to the killing of a newborn baby within the first 24 h (Lattanzi et al., 2020) mostly by his or her natural mother (Collins & Byard, 2014). Resnick's definition of neonaticide will be adopted within this current systematic review as it is still widely used in the literature.

Infanticide is defined by some authors as the act of killing an infant within the first year of life by his or her parent(s) (Debowska et al., 2015; West, 2007), while for others the relationship between those who committed the act, and the infant is not specified and might include step‐parent(s) and other caregiver(s) (Flynn et al., 2009; Lattanzi et al., 2020). However, according to England's legal definition (Aho et al., 2017) infanticide involves only biological mothers who engage in the act while still recovering from giving birth or from pregnancy or lactation related issues or mental health issues such as postpartum psychosis where hallucinations or delusions might lead to infanticide (American Psychiatric Association, 2013). Infanticide in this review will be referred to as an act undertaken by natural mothers within the first 12 months of a child's life as this systematic review aims to include only natural mothers within the population studied.

Filicide defines the killing of a child within the first 18 years of age committed by his or her parent(s), step‐parent(s) or other caregiver(s) (Horsford, 2016; West, 2007) or committed only by parent(s) regardless of the child's age (Bourget et al., 2007; Lattanzi et al., 2020; Liem & Koenraadt, 2008). Some studies include both neonaticide and infanticide as subcategories (Liem & Koenraadt, 2008) of the term filicide. The term filicide will be used in this review to refer to children after 12 months of life.

## 3 AIMS OF THE REVIEW

Studies aiming to understand the experience of maternal child killing from the viewpoint of the women involved rarely analyse the three phenomena of neonaticide, infanticide and filicide in tandem. When discussing child killing, considering only a specific age group in review studies might be extremely challenging (Flynn et al., 2009) due to differing definitions included in the literature and no clear differentiation between the three categories in primary research. For the same reason, interpretation and comparison of the findings might appear contradictory (Dawson, 2015).

The current systematic review aimed to identify and synthesise evidence on the perspective of women directly involved in the complex phenomenon of neonaticide, infanticide or filicide from the evidence available on their unique point of view and lived experience.

## 4 METHODS

A qualitative evidence synthesis (QES) method was chosen for this review as it offers an approach to describe interviewee views, values and feelings in line with original meanings of the primary qualitative studies included (Booth, 2017). QES does not set out to undervalue the importance of a single person's voice, but operates on the basis that integrating a wider range of perceptions and views on the same phenomenon might offer richer, even more powerful, explanations of an experience (Booth, 2017; Flemming & Noyes, 2021). The decision to choose a QES was informed by the RETREAT framework criteria such as review question, epistemology, time, resources, expertise, audience and purpose, and type of data (Booth et al., 2018). Specifically, a thematic synthesis approach was chosen as it is well‐suited to the review question, quantity and type of data available timeframe for the review which was undertaken as part of a MSc programme and as this approach is recommended when data included are not thick enough to carry out more interpretative QES such as meta‐ethnography (Booth et al., 2018; Noyes et al., 2019; Thomas & Harden, 2008). In addition, it is less reliant on the epistemology underpinning the method of each qualitative study included and findings resulting from thematic synthesis can be used to “directly inform practitioners” (Booth et al., 2018; Noyes et al., 2019; Thomas & Harden, 2008).

In order to enhance transparency, this review conforms to the reporting guidance provided in the Enhancing Transparency in Reporting the Synthesis of Qualitative Research (ENTREQ) statement (Tong et al., 2012). The protocol was registered with Open Science Framework and is publicly available online (Milia & Noonan, 2021).

### 4.1 Literature search and selection

#### 4.1.1 Study search strategy

The PEO framework (Bettany‐Saltikov & McSherry, 2016) was used to develop the research question: What are the experiences and perspectives of women who have committed neonaticide, infanticide or filicide?:

**P**opulation—women

**E**xposure—neonaticide, infanticide or filicide

**O**utcome—experiences and perspectives

A preliminary scoping search of relevant databases—Medline (EBSCO), CINAHL Complete (EBSCO), EMBASE (Ovid), PsycINFO (EBSCO), Web of Science, Elsevier's Scopus and PROSPERO was conducted, and no current systematic reviews or registered review protocols were identified. Subsequently, five bibliographic databases were systematically searched (GM) from inception to 1 December 2020 and re‐ran on 17 April: Medline (EBSCO), CINAHL Complete (EBSCO), EMBASE (Ovid), PsycINFO (EBSCO) and Web of Science. In addition, as this review aims to comprehensively identify all eligible studies (Tong et al., 2012), forward reference checking and citation searching of the reference list of the included articles were completed. EndNote software was used to manage references (The EndNote Team, 2013).

A two‐strand concept search strategy was tailored for each database search (Booth, 2016) to include searching a combination of both index terms and keywords: neonaticide, infanticide or filicide combined with qualitative research methods such as grounded theory, observational study, interview, experiences, views and perceptions. The two concepts were entered into the databases, truncated where appropriate and combined using the Boolean operators “AND” and “OR.”

#### 4.1.2 Inclusion criteria

Primary qualitative studies published in the English language that focus on the perceptions and experiences of women engaging in neonaticide, infanticide or filicide were included.

#### 4.1.3 Exclusion criteria

Studies including neonaticide, infanticide or filicide cases committed by other family members or non‐biological mothers were excluded unless data on biological mothers were clearly distinguishable in the study's findings section. Studies where no clear distinction was made between attempted and committed acts were excluded. Studies using secondary data such as review and editorials were excluded on the basis that they were not suitable to answer the research question. Quantitative studies were excluded as this review's focus was exclusively on data obtained directly from participants through interviews and focus groups (Sandelowski et al., 2006).

#### 4.1.4 Study selection

**FIGURE 1:**
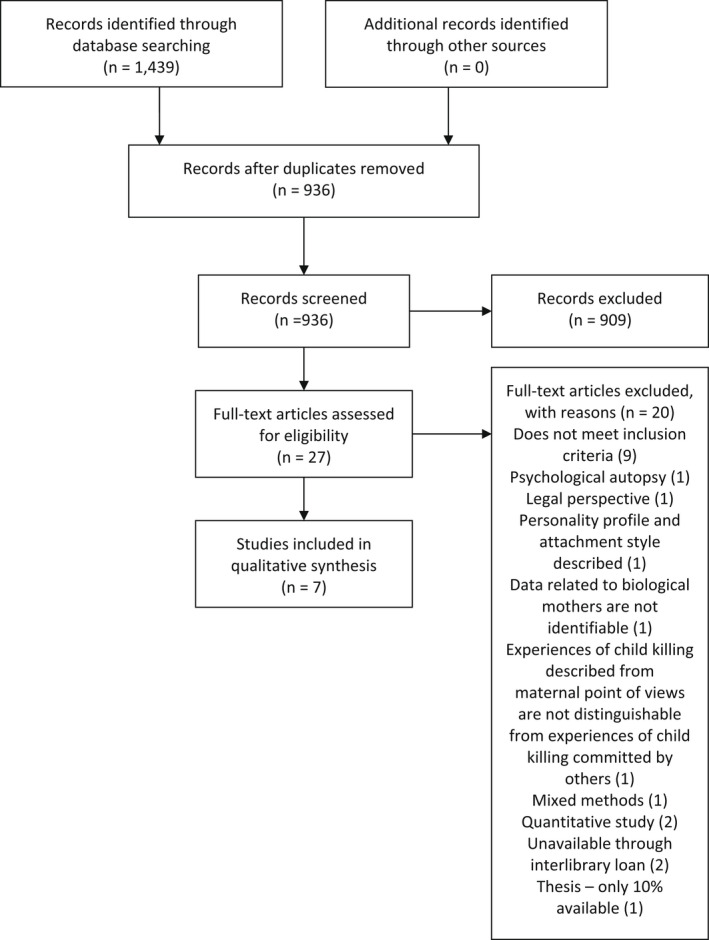
PRISMA flow diagram

A total of 1439 citations were retrieved from the combined searches of databases. Duplicate citations were removed through endnote and manually (*n* = 503) leaving 936 studies. In the first stage of the screening process, titles and abstracts were reviewed by the lead author according to the agreed inclusion and exclusion criteria using the Rayyan QCRI software programme (Ouzzani et al., 2016). Twenty‐seven studies selected for full‐text review were screened independently by both reviewers, and a total of seven papers reporting on the findings from five primary studies were included in the review. Discrepancies were resolved through discussion. Forward reference checking and citation searching of the reference list of included articles identified no further studies for inclusion. The Preferred Reporting Items for Systematic Reviews or PRISMA flow chart (Moher et al., 2009) illustrates the number of studies included and excluded at each review stage along with the rationale for exclusion (Figure 1).

#### 4.1.5 Quality assessment of studies

The included studies were critically appraised by the lead reviewer using the validated Critical Appraisal Skills Programme (CASP) tool (2018) which has been approved by the Cochrane Qualitative and Implementation Methods Group (Long et al., 2020; Soilemezi & Linceviciute, 2018). Appraisal decisions were cross‐checked by an independent reviewer (MN) for consistency and discrepancies resolved through discussion. As this review aims to offer an inclusive overview of the findings available in the literature, all studies were included irrespective of their quality rating (Butler et al., 2016). The main source of potential bias in included studies was uncertainty about the role of the researcher. The majority of the studies critically appraised were deemed high quality (Moodley et al., 2019; Riley, 2006; Stanton & Simpson, 2001, 2006; Stanton et al., 2000), while two were identified as medium quality (Gous & Roos, 2005; Razali et al., 2019).

### 4.2 Data extraction and synthesis

A data extraction tool (Table 1) was designed by the lead reviewer, piloted on three studies and revised accordingly and extracted data cross‐checked (GM and MN). The following data were extracted: study details (author, year, country); study aim(s), design and setting(s); methods of data collection and sample size; method of data analysis; findings (original themes identified); and quality appraisal (Aromataris & Munn, 2020; Butler et al., 2016).

**TABLE 1:** Data extraction table

Study details (author, year, country)	Study aim(s), design and setting(s)	Methods of data collection and sample size	Method of data analysis	Findings (original themes identified)	Quality appraisal
Gous and Roos (2005), South Africa	To reach an in‐depth understanding of the representations of a depressed woman who engaged in neonaticide. Single case study. Prison setting.	Documents from psychiatric hospitals, data from field notes, audiotaped and transcribed individual semi‐structured interviews. One participant.	The strategy described by Neuman (2000) was followed for data analysis.	Representations of the mother as mother‐of‐ herself‐as‐child Representations of herself‐ as‐a‐mother Representations of her children	A case study that addressed all of the CASP criteria.
Riley (2006), United States	To understand the personal interpretation that women who commit neonaticide place on this act and generate grounded theory on the subject of neonaticide. Prison setting.	Audiotaped individual semi‐structured interviews. Nine participant.	Thematic analysis.	Behavioural and psychological responses: Fear; Concealment; Emotional isolation; Denial; Dissociation; Act of neonaticide. Integral elements contributing to neonaticide: Cultural and religious issues; Family structure/relationship; Self‐concept/individual vulnerability	The majority of CASP criteria met. Further information on the process of informed consent required.
Stanton et al. (2000), New Zealand	To examine description of maternal filicide committed in the context of major mental illness using, as a frame of reference, a group of women involved in these acts. Naturalistic paradigm. Participants were accessed via their treating psychiatrists, and interviews were performed at their place of residence.	Audiotaped individual semi‐structured interviews. Six participants.	Thematic analysis.	The participants Experience before the event The children The event After the event	Further information required on data saturation and the relationship between researcher and participants.
Razali et al. (2019), Malaysia	To understand the meaning of and background of filicide from the perspectives of women convicted of filicide in Malaysia. Interpretive phenomenological analysis. Prisons and psychiatric institution s.	In‐depth interviews audiotaped if consent was given, otherwise detailed notes were taken by the author and verified with each woman. Nine participants.	Interpretative phenomenological analysis, narrative theory.	Living in a patriarchal society Poverty and limited opportunities Violence Isolation and lack of support Difficult motherhood Child death and punishment	Further information required on data saturation.
Moodley et al. (2019), South Africa	To examine women's perceptions of their offences and their treatment and rehabilitat ion in a South African context. Naturalistic paradigm. State inpatient or outpatients at Sterkfonte in Hospital.	Audiotaped and transcribed semi‐structured interviews. 11 participants.	Thematic analysis.	Negative feelings about being a state patient Mental instability during the incident Reflections post‐incident Closure Access to rehabilitation resources Access to support	CASP criteria addressed. Context of the study may have implications for the transferability of findings to other populations.
Stanton and Simpson (2001), New Zealand	The study focuses on the difficult area of child death misdiagnosed as sudden infant death syndrome (SIDS). The authors use the narratives of a woman who have committed such acts to enrich clinicians' knowledge of possible presenting features of the phenomenon. Naturalistic paradigm. Participant's place of residence.	Audiotaped and transcribed semi‐structured interviews. One participant.	Thematic analysis.	Planned and long‐awaited first pregnancy Onset of breathing problems The first murder Grieving for the first child's death Participant's second baby Assaults on other children	Single case study. Further information required on data saturation and the relationship between researcher and participant.
Stanton and Simpson (2006), New Zealand	To present a range of descriptions of recovery experiences derived from a qualitative study of women with mental health issues who have committed filicide. Naturalistic paradigm. Participants were accessed via their treating psychiatrists, and interviews were performed at the women's place of residence.	Audiotaped and transcribed semi‐structured interviews. Six participant.	Thematic analysis.	Managing the horror of the memories Language used to describe the event Forgiving themselves Role as mother Support Managing illness	Further information required on data saturation and the relationship between researcher and participants.

Thomas and Harden's (2008) thematic synthesis guided analysis. Key findings supported by relevant quotations were extracted from the findings section in each paper and imported onto the software programme NVIVO 12 (QSR International Pty Ltd., 2020). Line‐by‐line coding of meaningful data from the seven papers was undertaken. Both “first‐order” (participant interpretations of their experience) and “second‐order” concepts (author interpretation of participant experience) were inductively coded without applying any pre‐existing theory (Butler et al., 2016). Groups of descriptive codes were formed based on similarities between the codes. The contents of each of the groups of descriptive codes were iteratively explored, further refined and organized into a hierarchy of “descriptive themes and subthemes.” These were constantly reviewed against original themes to ensure consistency and to maintain original meanings. Analytical themes and subthemes were developed through an iterative process of merging descriptive themes with clear commonalities. The analysis and synthesis process was conducted by the lead reviewer with findings discussed and critically debated by both reviewers.

A challenge encountered during the process of analysis and synthesis of data was the use of inconsistent and universal terminology across different studies. In addition, the lack of indication of child age at time of death sometimes generated confusion and made it challenging to identify the similarities or differences among experiences and perceptions of women who engaged in these acts at various stages in a child's age. Therefore, the secondary aim of analysing experiences according to the child's age at time of death could not be achieved.

### 4.3 Reflexivity

Research is vulnerable to the influence of the researcher's gender, cultural and professional background along with their pre‐existing ideas and views on the chosen topic area (Barrett et al., 2020). To enhance this review's transparency, the lead author analysed her position as a reviewer. I am a midwife with a strong interest in perinatal mental health and identify as an “outsiders” towards both research participants and context (Barrett et al., 2020). I was motivated to undertake this review by a strong interest in gaining further understanding of the dynamics behind these acts and a firm belief in woman‐centred care led me to focus the review on understanding the positions and attitudes of women who had engaged in any of the three phenomena.

## 5 RESULTS

### 5.1 Characteristics of the selected studies

The median publication year of included articles was 2006 (range 2000–19). The studies were conducted in four countries: South Africa (Gous & Roos, 2005; Moodley et al., 2019), the United States (Riley, 2006), New Zealand (Stanton & Simpson, 2001, 2006; Stanton et al., 2000) and Malaysia (Razali et al., 2019). All seven papers reported on qualitative data and study design varied: single case study (Gous & Roos, 2005; Stanton & Simpson, 2001), grounded theory (Riley, 2006), naturalistic paradigm (Moodley et al., 2019; Stanton & Simpson, 2001, 2006; Stanton et al., 2000) and interpretative phenomenological analysis (Razali et al., 2019). Study settings were represented by prison institutions (Gous & Roos, 2005; Riley, 2006), participants' place of residence (Stanton & Simpson, 2001, 2006; Stanton et al., 2000), psychiatric hospitals (Moodley et al., 2019) and both prisons and psychiatric institutions (Razali et al., 2019). The included studies comprised a total of 36 participants. All seven papers based their results on data collected through semi‐structured in‐depth interviews and one (Gous & Roos, 2005) included documents from psychiatric hospitals where the participant was admitted and from field notes.

Riley (2006) explored women's experience of neonaticide. Experiences of women who undertook infanticide were explored in two studies reported across three papers (Gous & Roos, 2005; Stanton & Simpson, 2001; Stanton et al., 2000). The phenomena of infanticide and neonaticide were examined by Razali et al. (2019) whose study also included three acts of filicide where the biological mothers were incarcerated although the act was committed in association with or solely by their partner. Data collected on these women's experiences were excluded from this review as they did not meet inclusion criteria. Moodley et al.'s (2019) study on filicide did not specify child age at time of death. Stanton et al. (2000), Stanton and Simpson (2006) and Stanton and Simpson's (2001) findings are based on data collected from the same sample of participants and although child age at time of death ranged from a few weeks up to 7 years, a distinction between infanticide or filicide was not made.

Data on participant characteristics, background; major stressors; support system; clinical history; circumstances regarding pregnancy childbirth and postpartum; relationship with the child who died; child age; circumstances and motives related to child death are reported under three headings: cases of filicide; infanticide; and neonaticide.

### 5.2 Cases of neonaticide

The average age of women committing neonaticide was 25.8 (range 24–27.6) (Razali et al., 2019; Riley, 2006). Participants' background histories were characterised by poverty, social stigmatization and lack of support, unstable relationships with partners engaging in substance use or intimate partner violence (Razali et al., 2019; Riley, 2006). Some reported faith‐related concerns, individual vulnerability, superficial relationships with family and others, and growing up with abusive, neglectful and substance abusing parents or within the context of overreliance on family supports (Riley, 2006). Pregnancy in two cases resulted from rape, while in others it was characterised by fear of abusive partners or solitude having been abandoned by them after conceiving. Emotional isolation and fear of social and family disappointment led to pregnancy concealment and/or denial, no attendance at antenatal care culminating in unassisted childbirth (Razali et al., 2019; Riley, 2006). Neonaticidal acts were committed within the first 24 h after giving birth. Circumstances and motives of neonaticide were panic, denial (Razali et al., 2019; Riley, 2006) and dissociative response and/or fear of reaction of an abusive partner (Riley, 2006). Newborns died by suffocation or accidental death (Razali et al., 2019; Riley, 2006), death from an intentional fall (Razali et al., 2019) or drowning (Riley, 2006). Some participants reported the baby was stillborn or died shortly after childbirth (Razali et al., 2019; Riley, 2006).

### 5.3 Cases of infanticide

Women who carried out infanticide were described as in their 20s (Gous & Roos, 2005; Razali et al., 2019; Stanton & Simpson, 2001). Differences in background, major stressors, support structures and clinical history were highlighted among participants in all three studies. Lack of education, poverty, poor access to health care, domestic violence and substance use within the context of a patriarchal society, resentful and unstable relationships with unfaithful partners, growing up in a violent environment with abusive parents, inexistent support systems, were factors predominant among this group of women (Gous & Roos, 2005; Razali et al., 2019). In contrast, a more stable familial context and marital relationship were cited in another participant's life (Stanton & Simpson, 2001). Although backgrounds differed, all participants presented with mental health issues, namely personality (Stanton & Simpson, 2001), depressive (Gous & Roos, 2005; Razali et al., 2019) or psychotic disorders (Razali et al., 2019). Unwanted pregnancy, no access to abortion, lack of emotional involvement with the infant (addressed as “it”) and anger towards the woman's partner then projected towards the baby who resembled him, led to the woman's infanticidal act (Gous & Roos, 2005). Stanton and Simpson's (2001) paper describes a case study related to a woman who committed infanticide on two occasions. This woman described her first pregnancy as planned and strongly desired resulting in a deep bond with the infant. Infanticide for altruistic reason (mercy killing) was carried out due to the infant's breathing problems. The woman's second pregnancy was described as an attempt to save her relationship with her partner; however, this was still described as an “unwanted” pregnancy. Grief and guilt deriving from the first infanticide, together with worsening mental health postpartum and alcohol dependency, had led to the second infanticide (Stanton & Simpson, 2001). Auditory hallucinations and postpartum depression were the main motives considered in two further studies on infanticide (Gous & Roos, 2005; Razali et al., 2019). Median infant age at time of death was seven months (range 2.5–10). The method of killing was mainly suffocation (Gous & Roos, 2005; Razali et al., 2019; Stanton & Simpson, 2001) or injury due to an intentional fall (Razali et al., 2019).

### 5.4 Cases of filicide

The ages of women who committed filicide were indicated as “in their 20s” within two papers (Stanton & Simpson, 2006; Stanton et al., 2000) while in another the majority of participants ranged between 21 and 50 years (Moodley et al., 2019). Information on background, major stressors, support system and clinical history did not evidence a salient history of present or past abuse or social isolation. Circumstances regarding pregnancy, childbirth and postpartum were not discussed, with only one participant disclosing stress in pregnancy (Stanton et al., 2000). The relationship with the child victim was described as enjoyable before the onset of mental health issues (Stanton et al., 2000). Child age at time of death was not specified. Severe mental health issues were predominant among this group of women whose main reasons for engaging in filicide were represented by delusion‐based mercy killing or extension of suicide (Moodley et al., 2019; Stanton & Simpson, 2006; Stanton et al., 2000). Mental health conditions were either not identified before the death of the child or the condition had changed markedly prior to the filicide (e.g. history of depression with first manic episode) (Stanton et al., 2000). Killing methods were reported as stabbing, jumping with the child from a high place, setting fire to the house, drowning and suffocation (Moodley et al., 2019; Stanton & Simpson, 2006; Stanton et al., 2000).

## 6 THEMATIC SYNTHESIS

Three descriptive themes with associated subthemes were identified: (a) Experiences of women who undertook neonaticide; (b) Experiences of women who undertook infanticide; and (c) Experiences of women who undertook filicide. Further synthesis generated three analytical themes discussed below: (a) Not ready to be a mother; (b) Intentionality and premeditation in the context of trauma and mental health issues; (c) Sorrow of regret.

### 6.1 Theme 1: Not ready to be a mother

This theme discusses the several challenges that women who carried out neonaticide, infanticide or filicide appeared to encounter as they journeyed through pregnancy to motherhood. Data supporting this theme emerged from six of the papers in this synthesis (Gous & Roos, 2005; Moodley et al., 2019; Razali et al., 2019; Riley, 2006; Stanton & Simpson, 2001; Stanton et al., 2000) and are described in the following two subthemes.

#### 6.1.1 Sub‐theme 1: Difficulties in accepting the reality of pregnancy and motherhood

Women involved in neonaticide and infanticide found it difficult to accept the reality of their pregnancy, a feature that did not appear among filicidal women (Gous & Roos, 2005; Riley, 2006). Pregnancy denial and body dissociation seemed to only be related to women in involved in neonaticide. Participants described a process of disconnecting from the physical and emotional changes occurring in their bodies and what was happening along with a sense of necessary isolation (Gous & Roos, 2005; Riley, 2006):

I carried the pregnancy without acknowledging it at all. I just denied it the entire nine months. It never connected as being real. (Riley, 2006)

Both neonaticidal and infanticidal women appeared to share the same burden of “not being ready to be a mother.” Although some women within the neonaticide group experienced pervasive or intermittent denial, others were more aware of the reality of their pregnancy and consciously decided to conceal it by wearing loose clothing and avoiding any form of contact with family, friends and healthcare providers who might have detected the pregnancy (Razali et al., 2019; Riley, 2006):

I was constantly going into a form of isolation. I was driving away everybody that I needed to have near me. (Riley, 2006)

In cases where the women acknowledged an unwanted pregnancy, overwhelming feelings of fear towards their own parents, society and/or abusive partners led to both physical and emotional isolation and fear of rejection (Razali et al., 2019; Riley, 2006):

I was scared to death. He [partner] would've tossed me out on my ear. He was going downhill, drinking, getting into bar fights. I was afraid of him hurting me if he knew. (Riley, 2006)

I knew that this would be it. My mother would throw me out, nobody in the family would talk to me. (Riley, 2006)

Although only four women involved in infanticide were included in this review, contrasting experiences were displayed in two. While one woman felt inadequate in her role as a mother (Gous & Roos, 2005), another recounted feelings of joy after giving birth to a highly desired infant (Stanton & Simpson, 2001):

So I was not trying to make any sense at all. I was not ready to be a mother. I was not ready to be a mother. (Gous & Roos, 2005)

… this tiny little bundle, yeh, I couldn't believe it… It was a really neat feeling. Just having this little girl… all I’d ever wanted, a baby of my own. (Stanton & Simpson, 2001)

#### 6.1.2 Sub‐theme 2: Motherhood and living with mental health issues

A recurrent concept evident among women involved in infanticide or filicide was the impact of mental health issues on their role as mothers, women who committed neonaticide described psychological distress including dissociative responses, auditory hallucinations and history of trauma (Riley, 2006). Women across six papers experienced depressive or psychotic disorders (Gous & Roos, 2005; Moodley et al., 2019; Razali et al., 2019; Stanton & Simpson, 2001, 2006; Stanton et al., 2000):

I thought people were following me or vehicles were following me, or people were listening in to where I was staying or spying on me. It was terrible. (Stanton et al., 2000)

Some recounted the onset of mental health issues in pregnancy or postpartum (Razali et al., 2019; Stanton & Simpson, 2001; Stanton et al., 2000):

I was too far gone. I was just, like just not there, do you know what I mean… not mentally sick, but just, you know, couldn't talk. (Stanton & Simpson, 2001)

I remember thinking that the baby frowned a lot and that somehow, because I had been like really, really tired while I was pregnant and stressed, that I thought the baby had… caught the stress from me. (Stanton et al., 2000)

### 6.2 Theme 2: Intentionality and premeditation in the context of trauma and mental health issues

This theme focuses on the different contexts and situations faced by women who undertook neonaticide, infanticide or filicide and how those might have led them to the acts they carried out.

Women who engaged in acts of neonaticide, infanticide or filicide experienced intentions of killing in the context of mental health issues both in cases where premeditation was experienced or not (Moodley et al., 2019; Razali et al., 2019; Riley, 2006; Stanton & Simpson, 2001; Stanton et al., 2000).

In this review, most of the neonaticidal women experienced birthing their baby alone in their bedroom or bathroom, often without being fully conscious of what was happening in the context of denial of pregnancy that perdured throughout labour and childbirth (Razali et al., 2019; Riley, 2006). Women who committed neonaticide experienced panic and dissociative responses during unassisted birth and reported having no control over the events that happened and the deaths of their newborn babies were described as accidentally provoked (Razali et al., 2019; Riley, 2006):

It was like I was in a bubble watching all this happen, you know, that only I could see. It was like I was not in control of what was happening, like there was a separation of me from everything I did then. (Riley, 2006)

My intention at the time was to deliver her the best I could so that I didn't somehow hurt her. If I did do something to hurt her, it was never intentional. (Riley, 2006)

Similarly, findings across four studies suggested a lack of premeditation where women described committing infanticide or filicide within the context of delusion‐based altruistic acts and psychotic disorders with auditory hallucinations (Moodley et al., 2019; Razali et al., 2019; Stanton & Simpson, 2001; Stanton et al., 2000):

Then suddenly I went into my bedroom with my daughter, who was three months, and then when we got there I just started to have strange thoughts, like I don't know if it was voices or what, like I had to sacrifice my baby. That God wanted me to sacrifice my baby. (Moodley et al., 2019)

I thought she was going to go through what I had been through. I just thought that the devil was going to take [baby] in a cot death, that I had to save her and return her to the angels because if he took her, she'd go to Purgatory, she'd be stuck there forever. (Stanton et al., 2000)

Filicidal thoughts prior to the act were disclosed by a small number of women experiencing depressive disorders. Premeditation and intentionality were also evidenced in two studies where out‐of‐control anger and resentment led women experiencing mental health issues (depressive and personality disorder respectively) to infanticide (Gous & Roos, 2005; Stanton & Simpson, 2001):

I saw her like a stumbling block in front of me and that [it] was her I had to get rid of her. So I killed the baby and that was it. (Gous & Roos, 2005)

I knew exactly what I was doing, oh yes, I just, oh you know, I hated having to get up to feed her, I hated doing this, I hated doing that… Well, I thought, OK. I never got caught for [D1]’s death, I don't want this child, how am I gonna get rid of it, you know, so I smothered her the same way as I did with [D1]. (Stanton & Simpson, 2001)

### 6.3 Theme 3: Sorrow of regret

The third theme encompasses the experiences of women involved in neonaticide, infanticide or filicide after the deaths of their children. A recurrent concept in five papers of women who committed neonaticide, infanticide or filicide suggested that negative feelings such as grief, lack of self‐compassion and strong self‐hate were experienced after coming to terms with the act undertaken (Moodley et al., 2019; Riley, 2006; Stanton & Simpson, 2001, 2006; Stanton et al., 2000).

I blame myself every day for not making better choices. (Riley, 2006)

*Just feel regret*, *I wish I hadn't done what I did*. *Just regret*. (Moodley et al., 2019)

It was just really quite twisted it's just a real grief, which I hadn't dealt with. (Stanton & Simpson, 2001)

Regret for not asking for help appeared to characterize the women's lives after the deaths of their children.

Oh, why didn't I just pick up the phone and why didn't I just do this… I have been through that for the last three years. (Stanton & Simpson, 2006)

I really hate myself that I didn't get the right sort of help. (Stanton et al., 2000)

Negative feelings were mitigated in some filicide cases when the mental health issue that led to the act was recognised as a leading motive and subsequently acknowledged and accepted by the woman:

I was able to admit that I was severely depressed—I mean you know, and then to find that severe depression is a sickness is such a relief you know. (Stanton & Simpson, 2006)

On the contrary, one study on neonaticidal women suggested a lack of bonding or protective attachment with the newborn that was referred as “it” or “that thing” (Razali et al., 2019). Lack of grief appeared in another study on filicidal women who committed the act within the context of mental health issues:

When the police arrived they said: ‘he's giving mouth to mouth resuscitation.” I said “Oh no you can't bring her back’ cos I felt that all the good I’d done would be reversed. (Stanton et al., 2000)

## 7 DISCUSSION

Historically, women involved in neonaticide, infanticide or filicide are often defined as “mad” or “evil” by the public and the media. This is because their actions contradict those a maternal figure should present in our society: a protecting and loving figure whose priority is her children's physical and emotional wellbeing (Resnick, 2016). In considering the social stigma attached to the act of child killing, the findings aim to put the reader in the women's shoes, offering a different perspective on these apparently inexplicable and unacceptable acts by giving voice to these women and exploring their experience from their personal viewpoints.

Women involved in cases of neonaticide and infanticide described the struggle of “not being ready to be a mother” along with their difficulty in coming to terms with the reality of their pregnancies and their subsequent response of denial and/or dissociation. Although there is no official consensus on definitions of pregnancy denial, several authors have attempted to differentiate the concepts of pregnancy denial from concealment (Auer et al., 2019; Murphy Tighe & Lalor, 2016). While denial includes ignorance of a pregnancy that is not accepted as real, concealment is described as an active process of keeping the pregnancy secret by wearing loose clothes or avoiding social interaction that can be driven by fear of others (Murphy Tighe & Lalor, 2016). In line with Spinelli’s (2001) systematic investigation of 16 cases of neonaticide, pervasive and intermittent denial was observed in five and 11 women respectively. In Vellut et al.'s (2012) analysis of the relationship between neonaticide and denial of pregnancy, the phenomenon of pervasive denial was observed less often (two to three cases out of 32 women involved in neonaticide), while intermittent denial was more commonly experienced among women who alternately engaged in denial and conscious concealment by isolating themselves from others and avoiding antenatal care. In line with the findings identified in this review, concepts of “disabling fear,” especially towards family members, followed by concealment and isolation after finding out about an undesired pregnancy were highlighted.

In Shelton et al.'s (2011) review infanticidal and filicidal women described how living with the difficult reality of a psychotic or depressive disorder affected their motherhood. These findings support previous research that recounted mental health issues prevalent among infanticidal (Naviaux et al., 2020) and filicidal women (Flynn et al., 2013; Poteyeva & Leigey, 2018). However, in the current review, the majority of the infanticidal and filicidal women included were recruited from psychiatric settings; therefore, psychiatric population results were overrepresented. Contrasting results were found in the literature on mental health issues experienced by neonaticidal women. While Naviaux et al. (2020) supported the findings of scarce prevalence of mental health issues among women involved in neonaticide, Amon et al. (2020) reported that half of the 18 women included in their study experienced a mental health disorder at the time of neonaticide.

The lack of intentionality and premeditation was a recurrent concept observed in the majority of narratives by women who committed neonaticide, infanticide or filicide. These findings are in line with Amon et al.'s (2020) and Shelton et al.'s (2011) studies in which neonaticidal acts are described as carried out by women who have no intention of killing the baby. Dissociative responses followed by partial or total amnesia were described in Spinelli’s (2001) study of neonaticidal women who recounted “watching themselves” from outside while giving birth. This was similar to the accounts provided by women included in this review. Auditory hallucinations associated with dissociative responses were experienced only by one participant in our review, in contrast with Spinelli's (2001) sample where the majority of neonaticidal women experienced psychotic symptoms at the time of undertaking the act.

The findings based on women's narratives suggest that filicides were not committed deliberately due to the altered perceptions and mental status at time of the act. Altruistic filicide, according to Resnick's (1969) definition, is undertaken “out of love” by mothers who believe they are acting in the best interest of their children to relieve an often imagined suffering rather than harming them. On the other contrary, acutely psychotic filicide is committed due to delusions or auditory hallucinations with no apparent motive by a woman whose mental health status has been altered by a severe mental health disorder (Friedman et al., 2005). Although in earlier classifications altruistic and acutely psychotic filicide were presented as two separate underlying motivational factors, more recent literature has argued that both concepts should be studied in association as they are closely connected (Debowska et al., 2015).

Negative feelings such as intense grief and sense of guilt were recounted by the majority of women who committed neonaticide, infanticide or filicide after coming to terms with what they had done. In Mugavin's (2009) cross‐sectional exploratory study on risk factors that may contribute to fatal and non‐fatal abuse from the mothers' perspectives, women involved in filicide reported the death of their children as an extremely painful loss that provoked intense grief. This is in line with the findings of this review. However, lack of emotional attachment and a sense of relief after the child's death were reported by one neonaticidal and one filicidal woman in this review (Razali et al., 2019; Stanton et al., 2000).

The terminology used across different studies lacked consistency and was identified in some studies as quite judgmental. The stigma attached to these events is still strongly present in our society and evident in literature. Future research should consider adopting terminology that is more woman centred and mindful of the pain and struggles the women involved have experienced before and after engaging in child killing.

### 7.1 Limitations and implications for practice, education and research

The review included seven papers detailing five studies published in English which limits the review findings. Furthermore, two studies were case studies with one participant. The small numbers of participants across the studies are reflective of the sensitivity of the topic and rarity of phenomenon. Grey literature was not searched, and therefore, eligible studies may not have been identified. In addition, it is acknowledged that indexing of qualitative studies in databases varies and may impact on identification of studies for inclusion (Barroso et al., 2003).

This systematic review includes studies from South Africa, the United States, Malaysia and New Zealand, with an overrepresentation of the latter. The primary studies included women recruited from different settings offering a wide range of experiences and perspectives.

Representation of the three groups of women who engaged in neonaticide, infanticide or filicide are not exemplified equally within this review. Neonaticide, infanticide or filicide groups included 13, 4 and 17 women respectively. Furthermore, studies on filicide did not offer a clear or consistent definition of the term “filicide” or provide details on child age at time of death. Therefore, it is possible that some neonaticide or infanticide cases were included as a sub‐category and reported as filicide in the studies included. Hence, particular experiences, viewpoints and concepts may have been over‐ or under‐represented among the three groups of women. This highlights the need to use consistent and universally recognised definitions of neonaticide, filicide and infanticide where child age at time of death is clearly stated in studies that explore each of the phenomena. The interpretation of data may have been different if undertook from the perspective of a forensic mental health expert.

The findings of this review suggest that healthcare professionals need to attend to the unique experience of women. Healthcare professionals in contact with women during the perinatal period and beyond need to be aware of the profiles of vulnerable women and undertake holistic integrated assessments to identify the woman's personal context, changes in interpersonal relationships, social isolation or over reliance on family supports and changes in mental health status or new onset of mental health conditions. A markedly change in the woman's mental health condition prior to the filicide was reported by Stanton et al. (2000) and healthcare professionals need to consider this when assessing risk of filicide. Women require a clear plan of what to do if they feel overwhelmed with caring for a baby or child. Healthcare professionals involved with women in the perinatal period need to explore further women's expressions of “not being ready to be a mother” which for some women maybe pathological and require further assessment. Women need to be made aware of the support services pathways available to them during the perinatal period and beyond. Sexual and reproductive health education in secondary schools need to include a component that addresses concealed pregnancy outcomes in a sensitive manner.

The median publication year of the studies included is 2006, and the majority of studies are based on a limited number of participants. Furthermore, previous research may provide findings that are relevant to the social context of that time, and it is therefore desirable to carry out additional research aiming to update and expand on the literature in this area to attain a better understanding of the three phenomena. Moreover, short‐ and long‐term experiences of recovery of women after committing neonaticide, infanticide or filicide appear to be an understudied area of research underscoring the need for further primary qualitative studies to address this gap. Additional research involving women who avoided neonaticide, infanticide or filicide may inform prevention strategies.

## 8 CONCLUSIONS

The experiences of women involved in neonaticide, infanticide or filicide are scarcely discussed due to the phenomena's high sensitivity, rare incidence and issues that might be related to recruiting and interviewing the relevant women. On the contrary, these events are often sensationalised by the media when they occur. For these reasons women in need of help often fail to seek it our due to their fear of being judged. They are later often prey to feelings of guilt and regret for not having done so. Strategies should be devised and implemented to raise public awareness on the topic and to encourage women in these difficult situations to ask for help. The media could play a significant role in the dissemination of this information. In addition, the use of a more women‐centred, less judgmental terminology in literature—and the media—when these women are referred to and when cases of child killing are discussed publicly, would be of immense help in beginning the process of destigmatising these acts.

Despite its limitations, this review has provided insights into these infrequent but strongly taboo events in our society. Further research that explores women's experiences across cultures of committing and avoiding neonaticide, infanticide and filicide may add to the body of knowledge and support the identification of prevention strategies. A final recommendation includes using universally recognised definitions of neonaticide, infanticide or filicide where child age at time of death is stated clearly.

## 9 RELEVANCE STATEMENT

The Journal of Psychiatric and Mental Health Nursing was chosen as a journal for publication as its scope and publishing history well fitted this review title.

## ETHICS APPROVAL

This study is a review of existing literature and did not require ethics review.

## Data Availability

The data that support the findings of this study are available from the corresponding author upon reasonable request.
